# Deficiency in the Ubiquitin Conjugating Enzyme UBE2A in Alzheimer’s Disease (AD) is Linked to Deficits in a Natural Circular miRNA-7 Sponge (circRNA; ciRS-7)

**DOI:** 10.3390/genes7120116

**Published:** 2016-12-05

**Authors:** Yuhai Zhao, Peter N. Alexandrov, Vivian Jaber, Walter J. Lukiw

**Affiliations:** 1 LSU Neuroscience Center, Louisiana State University Health Science Center, New Orleans, LA 70112, USA; yzhao4@lsuhsc.edu (Y.Z.); vjaber@lsuhsc.edu (V.J.); 2 Department of Anatomy and Cell Biology, Louisiana State University Health Science Center, New Orleans, LA 70112, USA; 3 Russian Academy of Medical Sciences, Moscow 113152, Russia; wlukiw@yahoo.com; 4 Department of Ophthalmology, Louisiana State University Health Science Center, New Orleans, LA 70112, USA; 5 Department of Neurology, Louisiana State University Health Science Center, New Orleans, LA 70112, USA

**Keywords:** Alzheimer’s disease (AD), circular RNA (circRNA), genetic control, miRNA-7, proteasome, proteolysis, ubiquitin conjugating enzyme E2A (UBE2A)

## Abstract

Our understanding of the highly specialized functions for small non-coding single-stranded RNA (ssRNA) in the transcriptome of the human central nervous system (CNS) continues to evolve. Circular RNAs (circRNAs), a recently discovered class of ssRNA enriched in the brain and retina, are extremely stable and intrinsically resilient to degradation by exonuclease. Conventional methods of ssRNA, microRNA (miRNA), or messenger RNA (mRNA) detection and quantitation requiring free ribonucleotide ends may have considerably underestimated the quantity and significance of CNS circRNA in the CNS. Highly-specific small ssRNAs, such as the ~23 nucleotide (nt) *Homo sapien* microRNA-7 (hsa-miRNA-7; chr 9q21.32), are not only abundant in the human limbic system but are, in addition, associated with a ~1400 nt circRNA for miRNA-7 (ciRS-7) in the same anatomical region. Structurally, ciRS-7 contains about ~70 tandem anti-miRNA-7 sequences and acts as an endogenous, anti-complementary miRNA-7 “sponge” that attracts, binds, and, hence, quenches, natural miRNA-7 functions. Using a combination of DNA and miRNA array technologies, enhanced LED-Northern and Western blot hybridization, and the magnesium-dependent exoribonuclease and circRNA-sensitive probe RNaseR, here we provide evidence of a significantly misregulated ciRS-7-miRNA-7-UBE2A circuit in sporadic Alzheimer’s disease (AD) neocortex (Brodmann A22) and hippocampal CA1. Deficits in ciRS-7-mediated “sponging events”, resulting in excess ambient miRNA-7 appear to drive the selective down-regulation in the expression of miRNA-7-sensitive mRNA targets, such as that encoding the ubiquitin conjugating enzyme E2A (UBE2A; chr Xq24). UBE2A, which normally serves as a central effector in the ubiquitin-26S proteasome system, coordinates the clearance of amyloid peptides via proteolysis, is known to be depleted in sporadic AD brain and, hence, contributes to amyloid accumulation and the formation of senile plaque deposits. Dysfunction of circRNA-miRNA-mRNA regulatory systems appears to represent another important layer of epigenetic control over pathogenic gene expression programs in the human CNS that are targeted by the sporadic AD process.

## 1. Introduction

In eukaryotic cells the post-translational modification of end-stage proteins with ubiquitin is an important cellular regulatory mechanism for the targeting and shuttling of abnormal, short-lived proteins, transcription factors, and/or neurotoxic proteins destined for degradation and proteolysis into the ubiquitin-26S proteasome system [1,2,3]. As such, ubiquitin-mediated tagging, trafficking, and elimination of “waste” proteins plays a crucial role in cell-cycle regulation, DNA repair, cell growth, vesicular transport and immune function, and dysfunction of the ubiquitin-26S proteasome pathway is known to contribute to cancer, and immunological and neurodegenerative disorders [2,3,4,5,6,7]. The ubiquitination signaling pathway involves at least three categories of enzymes: a ubiquitin-activating enzyme UBE1, a ubiquitin-conjugating enzyme UBE2A, and a ubiquitin-protein ligase UBE3 [3,4,5,6]. The ubiquitin conjugating enzyme E2A (UBE2A) is part of an UBE2 enzyme group that catalyzes the transfer of ubiquitin from UBE1 to the active site cysteine of the UBE2A via a trans-thioesterification reaction and occupies a central regulatory position in the ubiquitination mechanism [3,4,5,6]. Interestingly, UBE2A (encoded at chr Xq24) is associated with neurological diseases that involve cognitive disruption, such as Alzheimer’s disease (AD), Parkinson’s disease (PD), mental retardation, X-linked syndrome, X-linked intellectual disability (Nascimento type), and other progressive, age-related neurodegenerative disorders [2,3,4,5,6].

Current studies involving bioinformatics analysis of microRNA-messenger RNA (miRNA-mRNA) coupling in the aging human brain indicate a significant miRNA-7-UBE2A-mRNA-3′-UTR interaction [7,8,9,10,11,12,13]. These studies, using multiple methods of analysis, subsequently uncovered a novel circular RNA (circRNA) for the miRNA-7 (ciRS-7) mechanism coupled to an ambient increase in miRNA-7 in the sporadic AD hippocampal CA1 region and superior temporal lobe neocortex (Brodmann A22) [7,8,9,10,11,12,13]; this may in part explain: (i) an expanding pathological role for increased miRNA-7 in inflammatory degeneration [14,15]; (ii) the down-regulation of UBE2A protein, a central effector in the ubiquitin-26S proteasome proteolysis and clearance system [3,4,5,6]; and (iii) the consequences of deficits in UBE2A and the ubiquitin-26S proteasome proteolysis, which are the accumulation and aggregation of brain waste products, such as amyloid proteins that are characteristic of the pathogenic lesions that progressively accumulate in the AD brain [16,17,18].

## 2. Materials and Methods

### 2.1. Reagents, Control, and Alzheimer’s Disease (AD) Brain Tissues

Except as indicated, reagents used in these experiments were obtained from independent commercial suppliers and were used without further purification. RNaseR, a magnesium-dependent hydrolytic 3′→5′ exoribonuclease that digests essentially all linear RNAs but not lariat or circular RNA structures, or double-stranded RNA with 3′-overhangs shorter than seven nucleotides was purchased from Epicentre (RNR07250 Illumina, Madison, WI, USA) and used according to established RnaseR protocols [5,10,19]. Alzheimer’s disease (AD) and age-matched control human temporal lobe and hippocampal CA1 were obtained from brain and tissue repositories, including the Institute for Memory Impairments and Neurological Disorders and the University of California at Irvine (UCI and our own archives); tissues were analyzed for total miRNA using miRNA arrays and LED-Northern blots and CFH abundance using Western blot analysis [11,12,13]. All AD and control brain samples were from adults; the mean (±1 SD) age of the control brain group (*N* = 6) was 71.6 ± 6.3 years and the mean postmortem interval (PMI; death to brain freezing interval) was 2.3 h; the mean age of the AD (*N* = 12) was 73.5 ± 6.1 years and the mean PMI was 2.2 h. There were no significant differences in the age, sex, or PMI between the AD and the control tissue groups.

### 2.2. Extraction of Total RNA and Protein and Quality Control

Total RNA and proteins were isolated simultaneously using TRIzol (Invitrogen, Carlsbad, CA, USA) and samples were enriched for small RNAs using spin columns, QIAzol lysis reagent, and RNase-free reagents and buffers (miRNeasy Mini Kit; Cat. No. 217004, Qiagen, Germantown, MD, USA). RNA quality was assessed using an Agilent Bioanalyzer 2100 (Lucent Technologies, Murray Hill, New Jersey, USA; Caliper Technologies, Mountain View, CA, USA). Typically, 1 µL of total RNA sample was loaded on an RNA chip (6000 Nano Labchip; Caliper Technologies, Mountainview, CA, USA) and analyzed for quality control; RNA integrity values were typically between 8.0 and 9.1. Protein concentrations were determined using the dotMETRIC microassay (sensitivity 0.3 ng protein/mL; Chemicon-Millipore, Billerica, MA, USA); Western blot and miRNA-mRNA complimentarity and prediction analysis was performed as previously described [14,15,17,18,20,21,22].

### 2.3. miRNA Array and LED-Northern Blot Analyses

miRNA labeling, hybridization, miRNA arrays, and reverse transcription polymerase chain reaction (RT-PCR) analysis were performed as described previously [15,16,17,18,20]. Samples were analyzed on miRNA arrays employing µParaflo® Microfluidic Biochip Technologies that interrogate the abundance and speciation of ~2650 human miRNAs (LC Sciences, Houston, TX, USA). LED-Northern dot-blot analysis was performed using a modified Bio-Dot microfiltration blotting device (LED = LNA, EDAC and DIG; LNA = locked nucleic acids; EDAC = 1-ethyl-3-3-dimethylaminopropyl carbodiimide; DIG = digoxigenin; detection limit = 0.05 fM of a single miRNA species; apparatus #170-6545, BioRad Life Science Research, Hercules, CA, USA) [10,11,12,13,16,20]. LED-Northern dot blots are a significant advancement over classical Northern blotting techniques because they utilize LNA-stabilized miRNAs or anti-miRNAs (AMs) covalently linked to a nylon-based membrane matrix (using EDAC) and are probed using DIG-labeled small RNAs with fluorescent reporters, thus generating higher specificity [16,20].

### 2.4. Western Blot Analysis of UBE2A and β-actin in AD and Control Tissues

Western immunoblots were performed for the quantification of UBE2A and β-actin protein in control and AD tissues using human-specific primary antibodies directed against the control protein marker β-actin (3598–100; Sigma-Aldrich, St. Louis, MO, USA) or human UBE2A (A-18; sc-10479: H-75; sc-30078; Santa Cruz Biotechnologies, Santa Cruz, CA, USA) or (PA5-29940; Invitrogen/Thermo Fisher Scientific, Waltham, MA, USA).

### 2.5. Statistical Analysis and Data Interpretation

**Figure 1:**
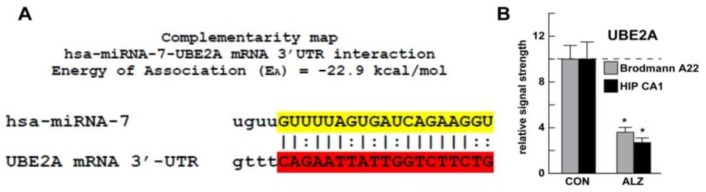
A strong miRNA-7-UBE2A mRNA 3’-UTR interaction. (**A**) Complimentarity map of human miRNA-7 and the UBE2A mRNA 3′-UTR—an “**|**” represents a hydrogen bond between the miRNA-7 and the UBE2A mRNA 3’-UTR and “**:**” represents a partial hydrogen bond; “seed” sequences are highlighted in yellow for miRNA-7 and in red for the UBE2A mRNA 3′-UTR; an energy of association (E**_A_**) between the miRNA-7 and the UBE2A mRNA 3′-UTR of −22.9 kcal/mol is very favorable thermodynamically; due to a deficit in ciRS-7 there are insufficient miRNA-7 sponging effects and an increase ambient miRNA-7 levels (Figure 2), resulting in a down-regulation in the expression of miRNA-7 targeted mRNA-3’-UTRs (such as UBE2A mRNA 3’-UTR) which is observed; other circRNAs may be involved; (**B**) levels of UBE2A protein in Brodmann area A22 and Alzheimer (ALZ) hippocampal CA1 compared to control (CON) brain postmortem samples; as in Figure 2, mean and one standard deviation are shown; UBE2A is observed to be reduced to 3.6- and 2.7-fold of controls, respectively, in Brodmann A22 (the superior temporal neocortex) and the hippocampal CA1 of AD brains and the results are highly significant; insufficient UBE2A might be expected to contribute to deficits in the ubiquitination cycle that helps to clear amyloid peptides via autophagy, thus contributing to amyloidogenesis in the AD brain; in (**B**) there were no significant differences in age, ApoE allele status, RNA quality (all RIN values were 8.1–9.0) or yield between the control (CON) or Alzheimer (ALZ) groups; control UBE2A levels in the Brodmann A22 (the superior temporal neocortex) and the hippocampal CA1 of AD were arbitrarily set to 10.0; a dashed horizontal line at 10.0 is included for ease of comparison; (* *p* < 0.001 (ANOVA).

All LED-Northern gel and miRNA-array data were analyzed as previously described [7,10,16,20]; statistical procedures for protein abundance (Western blot analysis) were performed using a two-way factorial analysis of variance (*p*, ANOVA) using programs and procedures in the SAS language (Statistical Analysis Institute, Cary, NC, USA). Only *p*-values less than 0.05 (ANOVA) were considered to be statistically significant. Complimentarity maps for miRNA-7-UBE2A-mRNA-3′-UTR (Figure 1) were generated using miRBASE [14]. Figures were generated using Excel 2011 (Microsoft, Redmond, WA, USA) and Photoshop CS2 version 9.0.2 (Adobe, San Jose, CA, USA).

## 3. Results

**Figure 2:**
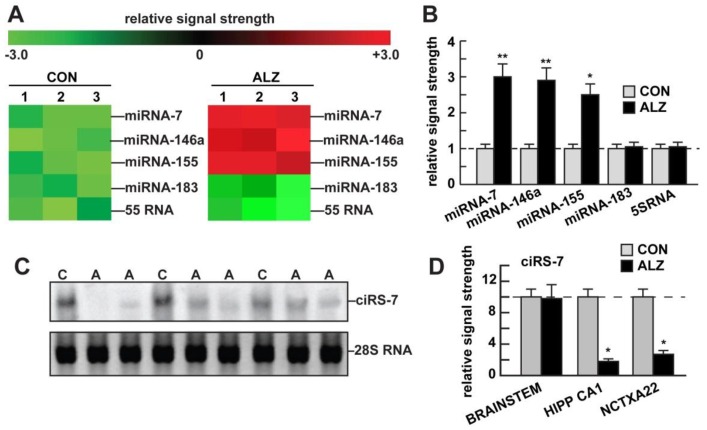
Increased miRNA-7 and deficits in ciRS-7 in AD. (**A**): up-regulated miRNA-7 in AD brain; lack of the miRNA-7 (ciRS-7) sponge by insufficient ciRS-7 suggests the miRNA-7 would be increased in the same brain regions where ciRS-7 is down-regulated as is observed; data in (**A**) quantified in bar graph format in (**B**); down-regulation of a family of miRNA-7-sensitive mRNA targets in the sporadic AD brain includes UBE2A; other mRNA targets may be involved; (**C**): detection of circRNA for miRNA-7 (ciRS-7) in sporadic AD and age-matched control hippocampal CA1 (control (CON) *N* = 4; AD (ALZ) *N* = 6]; in (C) the single upper ciRS-7 (~1400 nt) band contains ~70 selectively conserved miRNA-7 binding sites as previously described [8,10,11]; a lower 28S RNA served as an internal loading and reference control; all samples depleted of rRNA were treated with 50 units of RNaseR prior to electrophoresis see [8,10,23]); (**D**): AD ciRS-7 is significantly reduced to ~0.18-fold of control (CON) in AD (ALZ) hippocampal CA1 (HIPP CA1), to ~0.22 of control in the superior temporal lobe (Brodmann A22; NCTX A22) but not the brain stem (BRAINSTEM); this implicates loss of miRNA-7 sponge effects, and ambient up-regulation of miRNA-7 in at least two anatomical areas targeted by the AD process; there were no significant differences between age for control or AD tissues; (mean +/- one standard deviation (SD) = 71.6 ± 6.3 years (*N* = 6; control); 73.5 ± 6.1 years (*N* = 12; AD)); all AD cases were from moderate-to-advanced stages of AD; all postmortem intervals were 2.3 h or less; there were no significant differences in age, ApoE allele status, RNA quality (all RIN values were 8.1–9.0) or yield between the control or AD groups; Figure 1C has been redrawn in part from [10]; values in (B) and (D) are normalized to control levels; a dashed horizontal line at 1.0 or 10.0 is included for ease of comparison; (* *p* < 0.01, ANOVA); ** *p* < 0.001 (ANOVA).

Using a miRNA-array approach we quantified a significant increase in miRNA-7, miRNA-146a, and miRNA-155 in AD over an unchanging miRNA-183 or 5S RNA in the same sample analyzed; in this study ambient miRNA-7 was found to be increased to a mean (average) of about three-fold over age-matched controls (*p* < 0.001, ANOVA; Figure 2A,B). Predicted circular transcripts were found to consistently resist an RNaseR challenge; 30–35 ug total AD and control hippocampal CA1 or neocortical Brodmann A22 RNA were separated on agarose gels, transferred and probed with biotinylated or radiolabelled miRNA-7 probes, as previously described [8,10]. Detection was performed using a nonisotopic BrightStar BioDetect Kit (Ambion, Austin, TX, USA; detection limit ~100 fg) or by using standard autoradiography [9,16,18]. Since ciRS-7 (containing multiple anti-miRNA-7 sequences) appears to act as a type of “molecular sponge” for miRNA-7, we reasoned that miRNA-7 increases may be in part due to ciRS-7 deficits. Using a modified and highly-sensitive LED-Northern probing of AD and control brain hippocampal CA1 total RNA fractions (LED = locked nucleic acids—1-ethyl-3-3-dimethylaminopropyl carbodiimide (EDAC)—digoxigenin; LNA, EDAC, and DIG; detection limit = 0.05 fM of a single miRNA species; apparatus #170-6545, BioRad Life Science Research, Hercules, CA, USA) and a fluorescent- or radiolabeled miRNA-7 probe we detected ciRS-7 in human brain and found evidence for a significantly reduced abundance of ciRS-7 in AD to about 0.18-fold the abundance of age-matched controls (Figure 2C,D) [10,11,12,13,20]. In these studies we found no evidence for circRNAs encoding anti-miRNA-146a or anti-miRNA-155 sequences (data not shown). Using bioinformatics and complementarity alignment algorithms (see above) we found a strong potential interaction between *Homo sapien* (hsa) miRNA-7 and the hsa UBE2A mRNA 3′-UTR (Figure 1A). Subsequently, Western blot analysis of UBE2A in these same AD and control tissues showed a significant down-regulation in UBE2A protein to about 0.36-fold of controls in Brodmann A22 neocortex and 0.27-fold in the hippocampal CA1 region, two anatomical regions targeted by the AD process (Figure 1B).

## 4. Discussion

CircRNAs are generated (i) from both coding and noncoding exons, introns (including intron lariats); (ii) from 3′ and 5′ untranslated regions (UTRs) of mRNAs; (iii) from long non-coding intergenic sequences and pseudogenes; and (iv) from covalently-linked RNA ends produced in noncanonical splicing events, sometimes called “back-splicing” [19,21,22,24,25,26,27,28,29]. Perhaps the most intriguing feature of circRNAs are their lack of free ribonucleotide ends and their intrinsic circularity; their lack of free 3′ or 5′ termini have historically made circRNAs problematic to detect and quantify and, hence, their abundance, importance, and significance have only recently become appreciated [10,11,12,13,27,28,29]. ssRNA circularity bestows upon circRNAs a number of extremely interesting, novel and unique properties including: (i) the lack of free ribonucleotide ends that allows circRNAs to avoid exonucleolytic degradation by abundant cellular or nuclear RNA exonucleases and/or RNaseR makes them considerably more stable than linear mRNAs; (ii) the lack of a standard 3′-poly-A adenylation signal that is associated with most linear mRNAs; (iii) a greatly enhanced structural stability that enables circRNAs to prolong their capability for signal and information transfer from DNA to protein (note that these more stable circRNAs would be useful as novel biomarkers in disease versus, for example, miRNAs which have a generally shorter half-life); (iv) the capability and potential of circRNAs to be used in nuclear processes, such as the evolutionary ancient “rolling circle amplification (RCA)”; often used in prokaryotes, it has been recently shown that circRNAs are efficiently translated into functional proteins by RCA-directed mechanisms in living human cells [21]; and (v) that through RCA, circRNAs provide brain and retinal cells with a means to very rapidly express highly-specific spatiotemporal and genetic information, and particularly in anatomical localizations of the brain and retina, such as at synapses, where they may be enriched [19,21,25,26,27,28,29].

**Figure 3:**
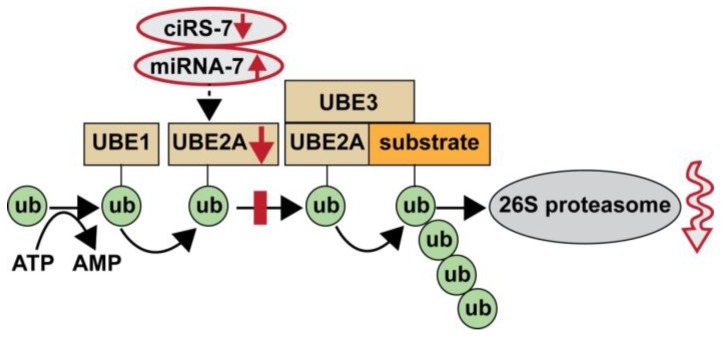
Highly schematicized illustration of deficits in the ubiquitination signaling pathway in AD that normally clears waste molecules (such as amyloid peptides) from the brain’s extracellular space: initially, a UBE1 enzyme activates, in an ATP-dependent manner, the 76 amino acid ubiquitin molecule by forming a high-energy thioester bond with ubiquitin; this E1-ubiqutin (UBE1) complex is next transferred to the ubiquitin conjugating enzyme (UBE2A); a ubiquitin ligase (UBE3) next positions the target substrate near the UBE2A enzyme allowing for the sequential transfer of ubiquitin; once a chain of four or more ubiquitin molecules is placed onto the substrate protein, the molecule is then targeted for proteolysis and degradation by the 26S proteasome; large and self-aggregating proteinaceous species (such as amyloids) once formed may be more difficult to ubiquitinate, sequester, and degrade [35]; a ciRS-7-driven miRNA-7-mediated lack of UBE2A near the center of this pathway would impair the trafficking of amyloid peptides and other neurotoxic end-stage molecules to the 26S proteasome complex [3,4,5,6,35,36]; note that in sporadic AD ciRS-7 is down-regulated (Figure 2) and ambient miRNA-7 is elevated, resulting in the down-regulation of UBE2A expression and an impairment at the UBE2A-ub stage (red rectangle) in the normal ubiquitination of end-stage molecules; this eventually leads to dysfunction and deficiency in the 26S proteasome system (downward wavy arrow at right).

While circRNAs involving mRNA-type sequences have been known for at least 25 years [9,19,21,22,24], circRNAs involving miRNA-type sequences in the brain was first described just three years ago in the advanced analyses of human transcriptome composition using RNA sequencing, RNaseR, and novel array-based detection technologies [10,11,12,23]. Recently, it has been discovered that various circRNAs can function as miRNA “sponges” that are also known as “competing endogenous RNAs” (ceRNAs) [30,31]. As the work in this paper indicates, circRNAs that contain anti-miRNA sequences in tandem may bind multiple miRNAs to repress their function(s) and competitively sequester miRNAs away from their natural mRNA 3′UTR targets [30,31,32,33,34]. Interestingly, the ubiquitin-conjugating enzyme UBE2A was the first ubiquitination component found to be targeted by a novel miRNA-7-UBE2A-mRNA-3’UTR-ciRS-7 mechanism that appears to normally shuttle neurotoxic and immunogenic amyloid peptides into proteolytic pathways via the ubiquitin-26S proteasome system, and, hence, clears the cytoplasm of end-stage toxic peptides and proteolytic debris (Figure 3) [10,11,12,34,35,36,37].

Lastly, miRNA-7 (miR-7) is a highly conserved, inducible miRNA abundant in the brain and retina of the human and murine CNS that displays restricted spatiotemporal expression during development, maturity, and disease, and the manipulation of miRNA-7 neurobiology has considerable diagnostic, prognostic, and therapeutic potential in human CNS health and disease [14,15]. Therapeutically, either alone or combined with other potent and versatile gene vector-encoded technologies or stabilized miRNA constructs, expression of custom-designed “anti-miRNA-7 sponges” could provide a new means of managing complex miRNA mixtures by “soaking up” selective and pathologically over-expressed miRNAs to regain homeostatic control of miRNA-7- and other miRNA-regulated gene expression.

In summary, the evidence presented here contributes at least four new pieces of information to the study of atypical RNA structures, circRNA, miRNA-7, and ciRS-7 in UBE2A function in the ubiquitin-26S proteasome system in the brain that appears to be significantly disrupted in AD, and perhaps other neurological diseases that involve progressive, age-related inflammatory neurodegeneration: 

(i) the UBE2A- and ubiquitin-mediated clearance of amyloid peptides and the degradation of unneeded, damaged, and/or neurotoxic proteins in brain cells appears to be regulated, in part, by gene products on two unlinked chromosomes: hsa-miRNA-7 encoded at chr 9q21.32 and UBE2A encoded at chr Xq24;

(ii) UBE2A occupies a central position in the ubiquitination signaling pathway; it is not currently well understood if the E1-ubiqutin (UBE1) complex or the ubiquitin ligase (UBE3) are also under miRNA regulation; if so, this would suggest that at least six individual gene products (three miRNAs and three UBE enzymes) may be required to moderate the functionality and mechanism of the ubiquitin-26S proteasome clearance system (Figure 3);

(iii) taken together these results suggest that the regulation of the ubiquitination cycle that normally orchestrates the clearance and degradation of amyloid peptides and/or damaged proteins by proteolysis via the 26S proteasome is genetically defective in sporadic AD brain; this deficiency has a strong potential to contribute to the inability to clear Aβ peptides from the cytoplasm, and this may have a bearing on the self-aggregation of Aβ peptides into higher order AD-related lesions and pathogenic amyloidogenesis; and

(iv) lastly, new discoveries of highly-specialized structures and functions for ssRNAs in the normal functioning of the eukaryotic genome, and whose activities are enriched in the human CNS, continues. It is perhaps surprising that evolutionarily ancient ssRNA structures and genetic mechanisms, such as “rolling circle amplification” (RCA) originally discovered in prokaryotes have persisted in evolution and still remain active in highly-evolved eukaryotic systems, such as those encountered in the human temporal lobe neocortex and hippocampal CA1 formation. Our understanding of ssRNAs, circRNAs, miRNAs, and miRNA-like molecules, and their enigmatic modes of complex interaction and dysfunction in health and disease, also continues to progress, and underscores the utilization of often non-conventional and novel genetic control and gene expression mechanisms in the homeostatic operation of the CNS.

## 5. Conclusions

In summary, we provide evidence for a novel and significantly misregulated ciRS-7-miRNA-7-UBE2A signaling circuit in sporadic AD neocortex (Brodmann A22) and hippocampal CA1. Deficits in ciRS-7-mediated “sponging events”, resulting in excess ambient miRNA-7 appear to direct the selective down-regulation in the expression of miRNA-7-sensitive mRNA targets, such as that encoding the ubiquitin conjugating enzyme E2A (UBE2A; chr Xq24). UBE2A that normally serves as a central effector in the ubiquitin-26S proteasome system is known to coordinate the clearance of amyloid peptides via proteolysis, and has been shown to be depleted in sporadic AD brain and, hence, contributes to amyloid accumulation and the formation of senile plaque deposits (Figure 3). Dysfunction of circRNA-miRNA-mRNA regulatory signaling appears to represent another important layer of epigenetic control over pathogenic gene expression programs in the human CNS that are targeted by the sporadic AD process.
